# Examining the moderated mediation effects of deep acting, personal accomplishment, and transformational leadership

**DOI:** 10.1186/s12912-026-04568-1

**Published:** 2026-03-21

**Authors:** Tung-sheng Kuo

**Affiliations:** Department of Business Administration, Nanhua University, Chiayi, 62249 Taiwan

**Keywords:** Deep acting, Transformational leadership, Personal accomplishment, Retention intention, Home care workers (HCWs), Taiwan

## Abstract

**Background:**

The rapid aging of the population has intensified demand for long-term care services, while persistent turnover among home care workers (HCWs) undermines service quality and sustainability. Emotional labor theory suggests that deep acting may improve retention, yet the mechanisms involving personal accomplishment and transformational leadership remain unclear.

**Methods:**

A cross-sectional survey of 386 certified HCWs in Chiayi, Taiwan measured deep acting, personal accomplishment, transformational leadership, and retention intention. Analyses included descriptive statistics, ANOVA, and PROCESS Model 8.

**Results:**

Direct effect analysis revealed that deep acting (B = 0.165, *p* = .009), transformational leadership (B = 0.160, *p* = .005), and personal accomplishment (B = 0.136, *p* = .017) significantly and positively predicted retention intention. Mediation and moderation effects were not supported. Demographic analyses indicated that older, married, and more experienced HCWs reported significantly higher retention intention. Female and long-tenured staff perceived stronger transformational leadership support.

**Conclusions:**

Deep acting and transformational leadership independently promote retention. Personal accomplishment also strengthens retention but does not mediate the effect of deep acting. The absence of moderation indicates that emotional regulation and leadership act as separate predictors. Workforce stability strategies should therefore cultivate genuine emotional engagement and supportive leadership as distinct organizational pillars.

## Background

Population aging is a critical global public health challenge, intensifying demand for long-term care. In Taiwan, 20.06% of the population was aged 65 or older in 2025, with Chiayi County reaching 24.11% [1]. In this context, workforce stability is paramount. Estimates from the Taiwan Ministry of Health and Welfare reveal a stark disparity: approximately 920,000 individuals require LTC services, yet fewer than 100,000 HCWs are available. This gap results in a service-to-client ratio exceeding 1:9, indicating a significant manpower imbalance that may undermine care quality and sustainability [2].

While substantive factors such as compensation and benefits play a crucial role [3], recent literature and meta-analyses suggest that psychological factors and emotional dynamics are often stronger predictors of retention than pay satisfaction alone [4, 5]. Specifically, excluding planned retirement, burnout and emotional exhaustion are primary drivers of turnover in healthcare settings [5]. Therefore, it is essential to understand the psychological mechanisms underlying HCWs’ retention, beyond financial incentives. Home care involves intensive emotional labor. Workers must often manage their emotions to align with organizational expectations. Among emotional regulation strategies, deep acting involves adjusting internal feelings to align with required displays, whereas surface acting involves superficial compliance [6]. Although deep acting is generally seen as a constructive strategy linked to lower burnout and higher retention than surface acting [7–10], findings remain inconsistent. Some studies indicate that even deep acting can consume significant cognitive resources, leading to burnout or turnover intention [11, 12] particularly in high-stress environments. This inconsistency highlights a critical research gap: under what conditions, and through what mechanisms, does deep acting effectively promote retention?

To clarify how deep acting relates to retention, this study focuses on personal accomplishment (sense of achievement). According to Herzberg’s Two-Factor Theory [13] and recent empirical studies, a sense of achievement is a vital intrinsic motivator that enhances job satisfaction and reduces turnover intention [14–16]. Within the nursing context, reduced personal accomplishment is a known predictor of turnover [17]. While Herzberg’s Two-Factor Theory identifies key motivators for retention, it often overlooks the dynamic psychological processes inherent in home care. To bridge this gap, this study integrates the Job Demands-Resources Model [18], providing a more robust framework to examine how personal resources (deep acting) and organizational support (transformational leadership) interact to foster personal accomplishment and long-term retention. Integrating the Job Demands-Resources model, this study conceptualizes deep acting as an adaptive emotional regulation strategy rather than a mere occupational demand. When HCWs successfully align their inner feelings with their work, this alignment acts as a resource investment that generates personal accomplishment—an intrinsic psychological resource—which in turn fosters stronger retention intention.

To resolve the inconsistencies regarding the effects of deep acting, transformational leadership is introduced as a critical boundary condition. Characterized by the ability to inspire followers through vision, individualized consideration, and motivation [19], this leadership style has been consistently linked to higher retention [20–23]. This study integrates Social Exchange Theory [24] with the resource substitution hypothesis from Conservation of Resources theory [25] to clarify these mechanisms. From a Social Exchange perspective, transformational leadership functions as a positive organizational input that elicits reciprocity. Simultaneously, based on the resource substitution hypothesis, it is argued that leadership support (a contextual resource) can substitute for individual resource investment (deep acting). Specifically, high transformational leadership may diminish the necessity for employees to rely solely on their own deep acting to generate a sense of accomplishment. Conversely, in the absence of strong leadership, deep acting becomes a critical compensatory resource. This study makes three contributions to the human resource management literature in long-term care. First, regarding mechanism clarification, it identifies personal accomplishment as a key mediator within the Job Demands-Resources framework, elucidating the pathway through which deep acting translates into retention. Second, by applying the resource substitution perspective, it resolves prior inconsistencies by establishing transformational leadership as a crucial boundary condition, explaining precisely when deep acting is most critical for retention. Finally, grounded in Social Exchange Theory, the study provides a robust contextual integration, explaining how organizational resources (leadership) and individual efforts (emotional labor) jointly contribute to workforce stability within Taiwan’s high-stress home care sector.

According to Emotional Labor Theory, deep acting requires employees to modify their inner feelings to match organizational display rules. This process reduces emotional dissonance and exhaustion, which in turn enhances job satisfaction and retention intention. Consequently, it is posited that deep acting serves as a critical predictor of retention.

- H1: Deep acting is positively related to retention intention.

Furthermore, personal accomplishment acts as a vital intrinsic motivator. Ac cording to the Two-Factor Theory, a sense of achievement leads to job satisfaction and reduces turnover intention. Although deep acting requires emotional effort, the successful management of emotions during caregiving interactions can foster a sense of professional efficacy and accomplishment. Therefore, personal accomplishment is expected to mediate the mechanism linking deep acting to retention.

- H2: Personal accomplishment mediates the relationship between deep acting and retention intention.

Finally, based on Social Exchange Theory, transformational leadership provides employees with external resources through inspirational motivation and individualized consideration. These leadership resources may interact with employees’ internal emotional regulation strategies (deep acting). Within the moderated mediation framework (PROCESS Model 8), this study hypothesizes that transformational leadership moderates the impact of deep acting on personal accomplishment (the first stage) as well as the direct effect of deep acting on retention intention (the direct path).

- H3: Transformational leadership moderates the relationship between deep acting and personal accomplishment.
- H4: Transformational leadership moderates the direct relationship between deep acting and retention intention.

**Fig. 1:**
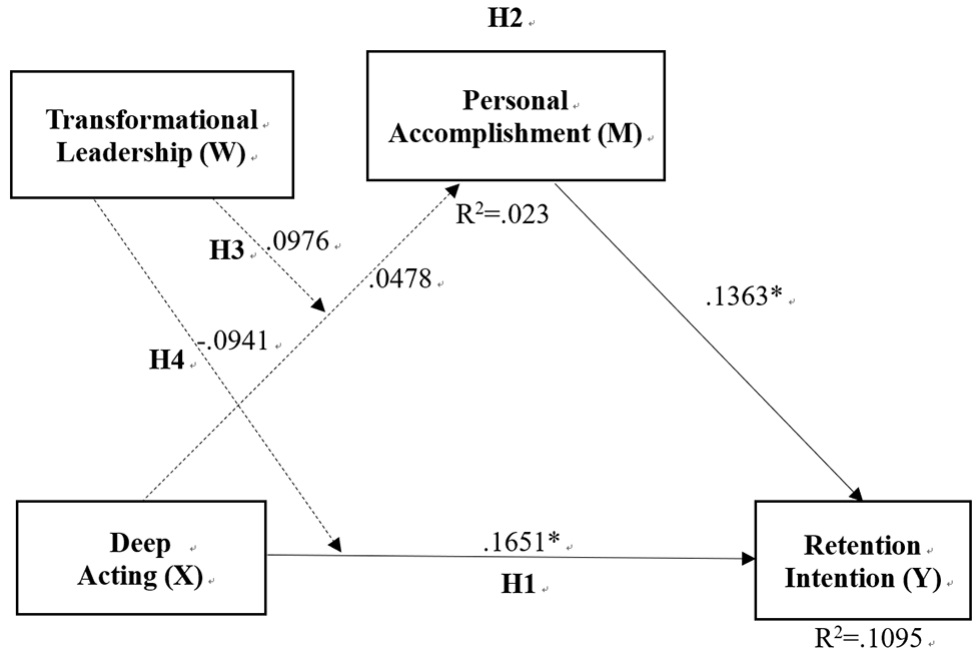
Statistical results of the hypothesized moderated mediation model. *Note*. N = 386. Values are unstandardized coefficients (B). Solid lines indicate *p* < .05; dashed lines indicate *p* > .05. * *p* < .05

To address these gaps, this study examines how deep acting influences retention intention through personal accomplishment, and whether transformational leadership moderates these relationships. The proposed conceptual model, illustrating the hypothesized paths (H1–H4), is presented in Fig. 1.

## Methods

### Design and settings

A cross-sectional quantitative research design was employed. The study was conducted in the Chiayi region, targeting certified HCWs. Data were collected between July and October 2022 using a standardized protocol. Administrative permission was obtained from the supervisors of 24 institutions to facilitate the research process. Chiayi was selected as the research site because its high elderly population proportion (22.41%) serves as a “leading indicator” for the challenges of an ultra-aged society in Taiwan [1]. The sample’s demographic profile—predominantly female (80.3%) and aged 35–64—highly aligns with national statistics of home-care workers, ensuring robust external validity [26].

### Participants and data collection

A purposive sampling method was used to collect data via an online Google Form accessed through a QR code. On-site visits were conducted to explain the study’s purpose directly to the participants. Before completing the survey, supervisors also provided a detailed explanation of the content to ensure clarity. A total of 386 valid responses were collected. To mitigate potential clustering effects, recruitment was diversified across the organizations, including 15 long-term care facilities, 6 hospitals, 2 nursing homes, and 1 health care association. To protect participant rights, it was explicitly stated that participation was entirely voluntary and anonymous. Electronic informed consent was obtained from each participant before accessing the questionnaire.

### Instruments

The questionnaire comprised five sections: demographic characteristics, deep acting, personal accomplishment, transformational leadership, and retention intention. Demographic data included seven items: gender, age, marital status, education level, tenure, monthly working hours, and monthly income.

Deep acting refers to the genuine regulation of inner emotions to meet organizational display rules [6]; it was measured with a 5-item scale adapted from Diefendorff et al. [27] and Groth et al. [28]. Personal accomplishment denotes the sense of achievement and meaning derived from work [13, 14]; it was measured with a 5-item scale adapted from Steger et al. [29] and Grant [30]. Transformational leadership reflects supervisors’ vision, individualized concern, and motivational support [19]; it was measured with a 7-item scale adapted from Kottke and Sharafinski [31] and Ystaas et al. [32]. Retention intention represents employees’ willingness to remain in their current position [33, 34]; it was measured with a 3-item scale adapted from Shahid [34]. All items were rated on a 5-point Likert scale (1 = Strongly disagree, 5 = Strongly agree). To ensure semantic equivalence within the traditional Chinese context, the initial instrument underwent a rigorous back-translation and revision process, facilitated by a panel of three experts: one hospital administrator and two university professors.

### Pilot study and psychometric evaluation

A pilot study of 50 samples was conducted to evaluate the psychometric properties of the instruments, following the framework proposed by DeVellis [35]. The item analysis focused on two primary dimensions: discriminative power and homogeneity. Results indicated that the critical ratio for all items across the four scales—Deep Acting, Personal Accomplishment, Transformational Leadership, and Retention Intention—attained statistical significance (*p* < .001), demonstrating robust discriminative power. To assess homogeneity, corrected item-total correlations were calculated, with all values significantly exceeding the recommended threshold of 0.30, thereby confirming high internal consistency. Furthermore, factor analysis supported the unidimensionality of each scale, as factor loadings primarily exceeded 0.45. These findings suggest the items reliably indicate their constructs; thus, the original instrument was retained without modification.

### Data analysis

Data analysis was performed using SPSS 22 and AMOS 17. Descriptive statistics, independent t-tests, and one-way ANOVA were used to describe the sample. Reliability was assessed using Cronbach’s α [36] and Composite Reliability (CR). Construct validity was evaluated using Confirmatory Factor Analysis (CFA). Mediation and moderation effects were examined using the PROCESS macro (Version 4.2, Model 8) developed by Hayes [37]. To ensure the robustness of the results, several demographic variables were controlled in the PROCESS Model 8 analysis. Specifically, age, tenure, monthly working hours, and monthly income were treated as continuous covariates. Education level was dummy-coded into three variables, with ‘Junior college/University’ (the largest group, *n* = 219) serving as the reference category to partial out potential confounding effects. Significance was determined using 5,000 bootstrap resamples with 95% bias-corrected confidence intervals (CI). To address potential common method bias resulting from the self-reported and cross-sectional nature of the data, Harman’s single-factor test was performed [38, 39].

### Ethical considerations

The study protocol was approved by the Research Ethics Committee of the Ditmanson Medical Foundation Chia-Yi Christian Hospital (IRB No. IRB2022050). The research was conducted in accordance with ethical standards to ensure participant anonymity and the confidentiality of the collected data.

## Results

### Reliability, validity, and common method bias

The measurement model’s reliability and validity were assessed with results summarized in Table 1. The Cronbach’s α coefficients (0.844–0.920) and Composite Reliability (CR) values (0.845–0.919) both indicate high internal consistency across all constructs. During the CFA refinement, one item from ‘Deep Acting’ and two from ‘Transformational Leadership’ were removed due to low factor loadings or collinearity. The final model demonstrated a good fit: χ^2^ / df = 2.800, GFI = 0.913, CFI = 0.956, and RMSEA = 0.068. Standardized factor loadings ranged from 0.552 to 0.960 (*p* < .001), and Average Variance Extracted (AVE) values ranged from 0.584 to 0.780, confirming satisfactory convergent validity. Furthermore, to address potential common method bias, the Harman’s single-factor test was performed by loading all study variables into an exploratory factor analysis. The results showed that no single factor emerged, and the first factor explained only 24.3% of the total variance, which is well below the 50% threshold. This confirms that common method bias was not a pervasive issue in this study and the observed variations were driven by the underlying constructs.

**Table 1:** Reliability and validity analysis

Construct	Shortened Label	Standardized Factor Loading	Cronbach’s α	CR	AVE
Deep Acting	• Positive mindset maintenance	0.552	0.844	0.845	0.584
	• Empathy cultivation	0.735			
	• Internal emotion regulation	0.898			
	• Client problem-solving	0.828			
Job Accomplishment	• Client/family recognition	0.604	0.920	0.919	0.701
	• Meaning in improvement	0.710			
	• Social contribution	0.919			
	• Delivering happiness	0.960			
	• Overall job achievement	0.931			
Transformational Leadership	• Proactive personal concern	0.739	0.902	0.903	0.654
	• Staff welfare focus	0.691			
	• Problem-solving support	0.824			
	• Individualized assistance	0.846			
	• Strength-based assignment	0.922			
Retention Intention	• Organizational commitment	0.958	0.903	0.913	0.780
	• Job-role persistence	0.915			
	• Value alignment	0.764			

Note. CR = Composite Reliability; AVE = Average Variance Extracted. Model Fit Indices: χ^2^ / df = 2.800; GFI = 0.913; CFI = 0.956; RMSEA = 0.068. All factor loadings are significant at *p* < .001

### Descriptive statistics of the sample

A total of 386 valid questionnaires were collected for this study. The demographic characteristics of the sample are summarized in Table 2. The workforce is predominantly female, accounting for 80.6% (*n* = 311) of the participants, which aligns with the gender distribution typically observed in the home care sector. In terms of age distribution, the majority of respondents were aged between 31 and 60 years, collectively representing 76.9% of the sample. The single largest age cohort was the 51–60 group (28.7%), suggesting that the primary workforce consists of middle-aged individuals. Regarding marital status, 68.4% of participants were married, followed by those who were single (23.3%), indicating that a significant portion of HCWs likely manage dual responsibilities of professional caregiving and family care. The educational profile shows that more than half of the participants (56.8%) held a junior college or university degree, while 30.3% completed senior high school. In terms of professional experience, 68.4% of the respondents had a tenure of less than 11 years, and 24.9% had been in the field for 11–20 years. The data regarding workload and compensation reveal a high-intensity work environment. Approximately 87.8% of HCWs worked more than 120 h per month, with nearly half (46.6%) exceeding 160 h. Despite these extensive working hours, monthly income for the vast majority (92.2%) was concentrated between USD 831 and 1,494. This mismatch between long working hours and low compensation underscores the socioeconomic vulnerability of the home care workforce.

**Table 2:** Demographic characteristics of the sample (*N* = 386)

Variable	Group	*n*	Percentage (%)
Gender	Male	75	19.4
	Female	311	80.6
Age (years)	18–30	72	18.7
	31–40	103	26.7
	41–50	83	21.5
	51–60	111	28.7
	Above 60	17	4.4
Marital Status	Married	264	68.4
	Single	90	23.3
	Divorced/ Widowed	32	8.3
Education	Junior high or below	24	6.2
	Senior high	117	30.3
	Junior college / University	219	56.8
	Graduate school and above	26	6.7
Tenure(years)	Under 11	264	68.4
	11–20	96	24.9
	Above 20	26	6.7
Monthly Working	< 121	47	12.2
Hours(hours)	121–160	159	41.2
	> 160	180	46.6
Average Monthly Income (USD)	< 831	14	3.6
	831–1162	215	55.7
	1163–1494	141	36.5
	> 1494	16	4.2

Note. Percentages are calculated based on valid samples. Income conversion is based on the exchange rate of September 2025 (1 TWD ≈ 0.03319 USD)

### Demographic differences in research constructs

To examine the variations in Deep Acting, Personal Accomplishment, Transformational Leadership, and Retention Intention across demographic profiles, independent samples *t*-tests and one-way ANOVAs were conducted, with Scheffe’s post-hoc comparisons performed for significant *F*-values (Table 3).

Regarding Deep Acting, significant differences were observed in age (*F* = 2.65, *p* = .049), marital status (*F* = 3.89, *p* = .021), and tenure (*F* = 5.76, *p* = .001). Post-hoc tests indicated that HCWs aged 51–60 and those with 11–20 years of experience engaged in deep acting significantly more frequently than the younger (18–30 years) and more junior (under 11 years) cohorts. Furthermore, married staff reported higher levels of deep acting than single staff. These results suggest that the internal alignment of emotions required for care work is positively associated with life experience and professional socialization.

For Personal Accomplishment, although the overall ANOVA models for age (*F* = 2.56, *p* = .045) and marital status (*F* = 3.03, *p* = .049) reached statistical significance, Scheffe’s post-hoc comparisons did not reveal specific pairwise differences between groups. Similarly, no significant variations were found for education, tenure, or income (*p* > .05). These findings imply that a sense of accomplishment in home care is a generalized sentiment derived from the intrinsic nature of the work rather than specific demographic factors.

Perceptions of Transformational Leadership varied significantly by gender and tenure. An independent samples *t*-test showed that female HCWs perceived significantly higher levels of leadership support than males (t = -2.36, *p* = .019). Furthermore, ANOVA results (*F* = 9.15, *p* < .001) indicated that senior HCWs—including both those with 11–20 years and over 20 years of tenure—rated their supervisors’ leadership significantly higher than those with less than 11 years of experience. This discrepancy may suggest a stronger relational exchange between supervisors and long-term or female staff.

Finally, Retention Intention demonstrated extensive demographic variability, with significant differences in age (*F* = 4.68, *p* = .003), marital status (*F* = 4.25, *p* = .015), education (*F* = 3.21, *p* = .042), tenure (*F* = 6.20, *p* = .002), and working hours (*F* = 3.51, *p* = .015). Post-hoc analysis emphasized a “stability effect”: HCWs who were older (51–60 years), married, and more senior (over 11 years of tenure) exhibited significantly higher retention intentions compared to their younger, single, and junior counterparts. Notably, although the overall F-tests for education and working hours were significant, Scheffe’s tests revealed no specific pairwise differences, suggesting these factors exert a more subtle, aggregate influence on workforce stability.

**Table 3:** Difference analysis of research constructs by demographic variables

Demographic Variable	Deep Acting	Personal Accomplishment	Transformational Leadership	Retention Intention
Gender	0.48	0.32	2.36*	0.82
Age	2.65*	2.56*	0.93	4.68**
Marital Status	3.89*	3.03*	0.26	4.25*
Education	2.14	0.15	0.43	3.21*
Tenure	5.76**	3.01	9.15***	6.20**
Working Hours	0.20	2.70	1.56	3.51*
Monthly Income	0.62	0.81	1.60	2.40

Note. Values for Gender are *t*-statistics from independent samples *t*-tests; values for all other variables are *F*-statistics from one-way ANOVA. Scheffe’s post-hoc tests were performed for significant *F*-values. *p < .05, **p < .01, ***p < .001

### Moderated mediation analysis

The moderated mediation model was examined using PROCESS v4.2 Model 8, with demographic covariates statistically controlled. The detailed estimation results are presented in Tables 4 and 5. Regarding the covariates, participants with an education level of junior high school or below reported significantly higher levels of personal accomplishment (B = 0.1085, *p* = .0393). Age also emerged as a marginally significant positive predictor of retention intention (B = 0.0663, *p* = .0574), suggesting that older employees might possess stronger intentions to remain in their positions. Other demographic variables did not demonstrate significant associations with either personal accomplishment or retention intention.

Regarding the focal predictors, deep acting exerted a significant positive direct effect on retention intention (B = 0.1651, *p* = .0095), implying that employees who engage in genuine emotional regulation are more inclined to continue their employment. Transformational leadership also showed a significant positive main effect on retention intention (B = 0.1599, *p* = .0052), underscoring the independent contribution of supportive leadership behaviors to employee retention. Additionally, personal accomplishment was a significant positive predictor of retention intention (B = 0.1363, *p* = .0176), highlighting the role of perceived achievement in sustaining the workforce.

The hypothesized mediating pathway was not supported. Specifically, deep acting did not significantly predict personal accomplishment (B = 0.0478, *p* = .4033), and the interaction between deep acting and transformational leadership on personal accomplishment was not significant (B = 0.0976, *p* = .3666). Similarly, the interaction between deep acting and transformational leadership on retention intention was not statistically significant (B = -0.0941, *p* = .4316), indicating that the impact of deep acting on retention intention does not vary significantly across different levels of transformational leadership.

As shown in Table 5, the conditional indirect effects through personal accomplishment were consistently non-significant across all levels of transformational leadership, as the bootstrapped 95% confidence intervals included zero. Finally, the index of moderated mediation was non-significant (Index = 0.0133, Boot SE = 0.0165, 95% CI [-0.0160, 0.0515]), confirming that transformational leadership did not significantly moderate the indirect effect of deep acting on retention intention via personal accomplishment.

Overall, deep acting, personal accomplishment, and transformational leadership were independently associated with higher retention intention. Contrary to expectations, however, the proposed moderated mediation model was not supported, as deep acting did not significantly predict personal accomplishment, and transformational leadership did not moderate either the direct or indirect pathways.

**Table 4:** Results of moderated mediation analysis (PROCESS Model 8, *N* = 386)

Outcome / Predictors	B	SE	t	*p*	95% CI
**Model 1: Personal Accomplishment (M)**					
Constant	4.224	0.1456	29.0107	0	[3.9377, 4.5102]
Deep Acting (X)	0.0478	0.0572	0.8367	0.4033	[-0.0646, 0.1602]
Transformational Leadership (W)	-0.0431	0.0514	-0.8384	0.4023	[-0.1442, 0.0580]
Interaction (X × W)	0.0976	0.108	0.904	0.3666	[-0.1147, 0.3099]
Covariates					
Age	-0.0169	0.0314	-0.5377	0.5911	[-0.0787, 0.0449]
Education (Junior high or below)^a^	0.1085	0.0525	2.0678	0.0393*	[0.0053, 0.2117]
Education (Senior high)^a^	-0.0344	0.1251	-0.2753	0.7833	[-0.2804, 0.2115]
Education (Graduate and above)^a^	0.0442	0.0708	0.6243	0.5328	[-0.0951, 0.1835]
Tenure	0.0268	0.1140	0.2348	0.8145	[-0.1973, 0.2508]
Monthly Working Hours	0.0555	0.0514	1.0801	0.2808	[-0.0456, 0.1566]
Monthly Income	-0.0303	0.0574	-0.5273	0.5983	[-0.1432, 0.0826]
**Model 2: Retention Intention (Y)**					
Constant	3.0654	0.2902	10.5639	0	[2.4948, 3.6360]
Deep Acting (X)	0.1651	0.0633	2.608	0.0095**	[0.0406, 0.2895]
Personal Accomplishment (M)	0.1363	0.0571	2.3855	0.0176*	[0.0239, 0.2487]
Transformational Leadership (W)	0.1599	0.0570	2.8081	0.0052**	[0.0479, 0.2719]
Interaction (X × W)	-0.0941	0.1196	-0.7873	0.4316	[-0.3293, 0.1410]
Covariates					
Age	0.0663	0.0348	1.9059	0.0574	[-0.0021, 0.1347]
Education (Junior high or below)^a^	0.0202	0.0584	0.3461	0.7295	[-0.0946, 0.1351]
Education (Senior high)^a^	0.141	0.1384	1.0188	0.309	[-0.1312, 0.4132]
Education (Graduate and above)^a^	0.0813	0.0784	1.0367	0.3006	[-0.0729, 0.2355]
Tenure	0.1204	0.1261	0.9549	0.3403	[-0.1275, 0.3684]
Monthly Working Hours	0.0578	0.0570	1.0152	0.3107	[-0.0542, 0.1699]
Monthly Income	0.002	0.0636	0.0309	0.9754	[-0.1230, 0.1269]

Note. B = unstandardized regression coefficient; SE = standard error; CI = confidence interval

Model 1: R^2^=0.0235, F(10, 375) = 0.9042, *p* = .5293. Model 2: R^2^=0.1095, F(11, 374) = 4.1802, *p* < .001

*p < .05, ** *p* < .01. Results estimated using PROCESS Model 8. ^a^ Reference group: Junior college / University

**Table 5:** Conditional direct and indirect effects of deep acting on retention intention at levels of transformational leadership

W Level	Direct Effect	SE	t	*p*	95% CI	Indirect Effect	Boot SE	95% Boot CI
Low (-1 SD: 3.40)	0.2178	0.0896	2.4301	0.0156	[0.0416, 0.3940]	-0.0009	0.0118	[-0.0253, 0.0236]
Moderate (Mean: 4.00)	0.1651	0.0633	2.6080	0.0095	[0.0406, 0.2895]	0.0065	0.0089	[-0.0088, 0.0266]
High (+ 1 SD: 4.60)	0.1124	0.0946	1.1881	0.2355	[-0.0736, 0.2983]	0.0140	0.0138	[-0.0086, 0.0465]
Index of Moderated Mediation	0.0133						0.0165	[-0.0160, 0.0515]

Note. Conditional effects estimated at low (-1 SD), mean, and high (+ 1 SD) levels of transformational leadership. Bootstrapped confidence intervals are based on 5,000 resamples. B = unstandardized regression coefficient; SE = standard error; Boot SE = bootstrapped standard error; CI = confidence interval. * *p* < .05, ** *p* < .01

## Discussion

This study examined the psychological and organizational factors associated with HCWs’ retention intention in Taiwan. Consistent with Hochschild’s emotional labor theory [6], the analysis confirmed that deep acting has a significant positive direct effect on retention intention. This finding supports prior research [7, 10], suggesting that genuine emotional engagement helps employees align with organizational display rules, ultimately serving as a protective resource against turnover.

Transformational leadership also exhibited a robust positive main effect on retention intention, echoing findings that emphasize the role of supportive leadership in enhancing workforce stability [20, 23]. In the home care context, where workers often operate independently, the psychological presence of an inspirational leader serves as a crucial anchor for retention. Furthermore, personal accomplishment was found to significantly predict retention intention, reinforcing the Job Demands-Resources model [18], which highlights intrinsic motivators as critical drivers of workforce stability.

### Implications of non-significant mediation and moderation

Regarding the hypothesized moderated mediation model, the results indicated that the interaction between deep acting and transformational leadership did not reach statistical significance. In contrast to some prior studies [9, 17] that reported a positive association between deep acting and personal accomplishment, our findings diverge from this pattern. Related research has also emphasized that emotional labor strategies such as deep acting can reduce burnout and enhance job satisfaction, thereby indirectly supporting accomplishment and retention [40]. Nonetheless, in Taiwan’s home-care context, deep acting appears to function primarily as a direct stabilizing mechanism for retention, rather than indirectly through personal accomplishment. The hypothesized mediation was not supported, suggesting that deep acting, although beneficial for retention intention, may serve more as a coping mechanism that sustains employees’ willingness to remain in their roles rather than fostering stronger feelings of accomplishment. In the demanding environment of home care, the effort required for deep acting may consume significant emotional and cognitive resources without immediately generating accomplishment. In such isolated environments, emotional regulation sustains relationship quality and retention intention directly, unless reinforced by external recognition or peer support. Similarly, the non-significant moderation effect of transformational leadership implies that leadership behaviors and emotional regulation function as independent predictors of retention rather than interacting with one another. The positive association of deep acting with retention intention remains consistent regardless of the level of transformational leadership, and leadership itself contributes separately to retention. This highlights the importance of viewing leadership and emotional regulation as parallel mechanisms that both strengthen workforce stability but do not necessarily amplify each other’s effects.

### Demographic and contextual factors

Demographic differences further contextualize these findings. While preliminary analyses suggested variations in marital status, the multivariate analysis revealed that education level—specifically having a junior high school education or below—was a significant predictor of personal accomplishment (B = 0.1085, *p* = .0393). Interestingly, external conditions such as working hours and income did not significantly differentiate retention intention in the final model, implying that psychological and relational factors outweigh material rewards for HCWs in Taiwan [2, 8].

In summary, this study clarifies that deep acting, transformational leadership, and personal accomplishment independently enhance retention. The absence of significant moderation underscores that organizations must cultivate both internal emotional skills and external leadership quality as separate but equally vital pillars of their human resource strategy.

### Managerial implications

Given the unique context of Taiwan’s home-care sector, where workers frequently operate in social and geographical isolation, generic organizational support is often insufficient. Because deep acting, transformational leadership, and personal accomplishment independently predict retention, organizations should adopt a multi-faceted intervention strategy. Since the hypothesized mediation and moderation effects were not supported, emotional regulation and leadership development must be treated as independent pillars of workforce stability rather than as mutually reinforcing or substitutive mechanisms. First, because deep acting does not naturally translate into personal accomplishment, agencies should proactively bridge this psychological gap by formalizing peer-validation protocols and recognition systems. This reframes emotional regulation as a core professional competency rather than a solitary burden. Second, transformational leadership training should be prioritized for supervisors, focusing on individualized consideration and emotional support. Structured check-ins and on-site emotional debriefing sessions can provide direct support that complements workers’ emotional labor. Finally, interventions should be tailored to diverse educational backgrounds, ensuring that professional mastery and caregiving contributions are consistently recognized across the workforce. By cultivating both genuine emotional engagement and supportive leadership styles in parallel, organizations can strengthen workforce stability in a sector characterized by high stress and isolation.

### Limitations and future research

This study has several limitations that should be acknowledged. First, the use of a cross-sectional design restricts the ability to draw causal inferences between deep acting, personal accomplishment, transformational leadership, and retention intention. Future research could employ longitudinal or experimental designs to better capture the dynamic processes underlying emotional labor and leadership. Second, the data were collected from HCWs in Chiayi, Taiwan, which may limit the generalizability of the findings to other regions or cultural contexts. Comparative studies across different healthcare systems and cultural settings would help validate the robustness of these results. Third, the reliance on self-reported measures raises the possibility of common method bias, although Harman’s single-factor test suggested that this was not a pervasive issue. Future studies could incorporate multi-source data, such as supervisor ratings or objective performance indicators, to strengthen validity. Finally, while the study focused on deep acting and transformational leadership, the non-significant mediation and moderation effects suggest that the pathways to retention are more complex than initially hypothesized. Future research should therefore examine additional psychological and situational factors—such as resilience, perceived organizational justice, or client relationship characteristics—that may clarify how emotional labor translates into personal accomplishment and long-term commitment. By broadening the scope of predictors and integrating multi-level perspectives, future studies can provide a more comprehensive understanding of workforce stability in the home-care sector.

## Conclusion

This research clarifies the factors associated with retention intention among HCWs in Taiwan. The analysis confirms that while deep acting, transformational leadership, and personal accomplishment are all essential for retention, they do not function in a synergistic or moderated fashion. Instead, they act as independent pillars of workforce stability. A primary contribution of this study is the confirmation that emotional effort, leadership quality, and intrinsic achievement are distinct pathways to employee commitment. Without formal organizational recognition and supportive leadership, deep acting remains a solitary burden of labor. Consequently, retention strategies must move beyond generic training toward on-site debriefing and peer-validation protocols. By redefining emotional regulation as a professional mastery and fostering a supportive leadership environment, agencies can transform home care into a sustainable and rewarding career. These findings also highlight the need for future research to explore additional psychological and contextual factors that may further clarify how emotional labor and leadership contribute to long-term workforce stability in home care.

## Data Availability

The datasets used and/or analysed during the current study are available from the corresponding author on reasonable request.

## Abbreviations

ANOVA: Analysis of variance
CFA: Confirmatory Factor Analysis
HCWs: Home care workers
IRB: Institutional Review Board

## References

[1] Lai YZ. Taiwan becomes super-aged society with over 20% of population aged 65 or older; Taipei records highest proportion at 24.18% [Internet]. Taipei: Central News Agency; 2026 Jan 9 [cited 2026 Jan 30]. Available from: https://www.cna.com.tw/news/aipl/202601090098.aspx (In Chinese).

[2] Long-term Care Center, National Taipei University of Nursing and Health Sciences. [Long-term care demand reaches 920,000 in super-aged society, highlighting severe caregiver shortages]. 2025 Feb [cited 2026 Jan 30]. Available from: https://longtermcare.ntunhs.edu.tw/application/view/news_content.php?id=3177 (In Chinese).

[3] Jiang L, Wider W, Ye G, Tee M, Hye AKM, Lee A, et al. Exploring the factors of employee turnover intentions in private education institutions in China: a Delphi study. Cogent Bus Manag. 2024;11(1):2299834.

[4] Griffeth RW, Hom PW, Gaertner S. A meta-analysis of antecedents and correlates of employee turnover: Update, moderator tests, and research implications for the next millennium. J Manage. 2000;26(3):463–88.

[5] Muir KJ, Porat-Dahlerbruch J, Nikpour J, Leep-Lazar K, Lasater KB. Top factors in nurses ending health care employment between 2018 and 2021. JAMA Netw Open. 2024;7(4):e244121. 38592723 10.1001/jamanetworkopen.2024.4121 PMC11004833

[6] Hochschild AR. The managed heart: Commercialization of human feeling. Berkeley: University of California Press; 1983.

[7] Zhu T, Park SK, Tu R, Ding Y. Does emotional labor trigger turnover intention? The moderating effect of fear of COVID-19. Sustainability. 2023;15(21):15336.

[8] Cho YN, Rutherford BN, Friend SB, Hamwi GA, Park J. The role of emotions on frontline employee turnover intentions. J Mark Theory Pract. 2017;25(1):57–68.

[9] Ha DJ, Park JH, Jung SE, Lee B, Kim MS, Sim KL, et al. The experience of emotional labor and its related factors among nurses in general hospital settings in Republic of Korea: A systematic review and meta-analysis. Sustainability. 2021;13(20):11634.

[10] Kuo TS, Chu LC, Shih CL, Li YC, Kao PL. Emotional labor, job satisfaction, and retention among home care workers in Taiwan: A comprehensive analysis. Front Psychol. 2025;16:1545955. 40231001 10.3389/fpsyg.2025.1545955 PMC11994665

[11] Lim J, Moon KK. Exploring the effect of emotional labor on turnover intention and the moderating role of perceived organizational support: Evidence from Korean firefighters. Int J Environ Res Public Health. 2023;20(5):4379. 36901390 10.3390/ijerph20054379 PMC10002436

[12] Li Y, You H, Oh S. A study on the structural relationship between emotional labor, job burnout, and turnover intention among office workers in Korea: The moderated mediating effect of leader-member exchange. BMC Psychol. 2024;12:54. 38287452 10.1186/s40359-024-01545-8 PMC10826281

[13] Herzberg F, Mausner B, Snyderman BB. The motivation to work. New York: Wiley; 1959.

[14] Seifert TA, Perozzi B, Li W. Sense of accomplishment: A global experience in student affairs and services. J Stud Aff Res Pract. 2022;60(2):250–62.

[15] Metz JD. The impact of achievement motivation, job satisfaction and work-life balance among retail managers [dissertation]. Malibu (CA): Pepperdine University; 2018.

[16] Wartenberg G, Aldrup K, Grund S, Klusmann U. Satisfied and high performing? A meta-analysis and systematic review of the correlates of teachers’ job satisfaction. Educ Psychol Rev. 2023;35:114.

[17] Karimi L, Leggat SG, Bartram T, Rada J. The association between dimensions of professional burnout and turnover intention among nurses: A systematic review. J Nurs Manag. 2022;30(5):1234–46.

[18] Bakker AB, Demerouti E. The Job Demands-Resources model: State of the art. J Manag Psychol. 2007;22(3):309–28.

[19] Bass BM. Leadership and performance beyond expectations. New York: Free; 1985.

[20] AbdElhay ES, Taha SM, El-Sayed MM, Helaly SH, AbdElhay AS. Nurses retention: The impact of transformational leadership, career growth, work well-being and work-life balance. BMC Nurs. 2025;24:148. 39923025 10.1186/s12912-025-02762-1 PMC11807322

[21] Gyensare MA, Kumedzro LE, Sanda A, Boso N. Linking transformational leadership to turnover intention in the public sector: The influences of engagement, affective commitment and psychological climate. Afr J Econ Manag Stud. 2017;8(3):314–37.

[22] Saeed F, Jun Y. The impact of transformational leadership on employee turnover intention: The mediating and moderating role of affective organizational commitment and job embeddedness. Int J Manag Acc Econ. 2022;9(5):247–67.

[23] Xiong B, Wu X, Sui Q. The impact of transformational leadership on the turnover intention of the new generation of knowledgeable employees: A moderated mediation model. Front Psychol. 2023;13:1090987. 36778168 10.3389/fpsyg.2022.1090987 PMC9909403

[24] Blau PM. Exchange and power in social life. New York: Wiley; 1964.

[25] Hobfoll SE. Conservation of resources: A new attempt at conceptualizing stress. Am Psychol. 1989;44(3):513–24. 2648906 10.1037//0003-066x.44.3.513

[26] Ministry of health and welfare, statistics on long-term care services and gender analysis. 2023. Taipei: MOHW; 2024. Available from: https://www.mohw.gov.tw/dl-87229-ddde835f-db98-4b56-9186-36a505999965.html (In Chinese).

[27] Diefendorff JM, Croyle MH, Gosserand RH. The dimensionality and antecedents of emotional labor strategies. J Vocat Behav. 2005;66(2):339–57.

[28] Groth M, Hennig-Thurau T, Walsh G. Customer reactions to emotional labor: The roles of employee acting strategies and customer detection accuracy. Acad Manage J. 2009;52(5):958–74.

[29] Steger MF, Dik BJ, Duffy RD. Measuring meaningful work: The work and meaning inventory (WAMI). J Career Assess. 2012;20(3):322–37.

[30] Grant AM. The significance of task significance: Job performance effects, relational mechanisms, and boundary conditions. J Appl Psychol. 2008;93(1):108–24. 18211139 10.1037/0021-9010.93.1.108

[31] Kottke JL, Sharafinski CE. Measuring perceived supervisory and organizational support. Educ Psychol Meas. 1988;48(4):1075–9.

[32] Ystaas LMK, Nikitara M, Ghobrial S, Latzourakis E, Polychronis G, Constantinou CS. The impact of transformational leadership in the nursing work environment and patients’ outcomes: A systematic review. Nurs Rep. 2023;13(3):1271–90. 37755351 10.3390/nursrep13030108 PMC10537672

[33] Ribelin PJ. Retention reflects leadership style. Nurs Manage. 2003;34(8):18–9. 12888725 10.1097/00006247-200308000-00008

[34] Shahid A. Employee Intention to Stay: An Environment Based on Trust and Motivation. J Manag Res. 2018;10(4):57–71.

[35] DeVellis RF. Scale development: Theory and applications. 4th ed. Thousand Oaks, CA: Sage; 2016.

[36] Nunnally JC. Psychometric theory. 2nd ed. New York: McGraw-Hill; 1978.

[37] Hayes AF. Introduction to mediation, moderation, and conditional process analysis: A regression-based approach. 3rd ed. New York: Guilford Press; 2022.

[38] Podsakoff PM, MacKenzie SB, Lee JY, Podsakoff NP. Common method biases in behavioral research: A critical review of the literature and recommended remedies. J Appl Psychol. 2003;88(5):879-903 10.1037/0021-9010.88.5.879 14516251

[39] Howell JP, Dorfman PW, Kerr S. Moderator variables in leadership research. Acad Manage Rev. 1986;11(1):88-102.

[40] Brotheridge CM, Grandey AA. Emotional labor and burnout: comparing two perspectives of people work. J Vocat Behav. 2002;60(1):17–39. 10.1006/jvbe.2001.1815.

